# A Treatise on Human Physiology for the Use of Students and Practitioners of Medicine

**Published:** 1899-07

**Authors:** 


A Treatise on Human Physiology for the use of Students and Practitioners of Medicine.—By Henry C. Chapman, M. D., Professor of Institutes of Medicine and Medical Jurisprudence in the Jefferson Medical College of Philadelphia. New (2nd) edition, thoroughly revised. In one handsome octavo volume of 921 pages, with 595 engravings. Cloth, $4.25, net; leather, $5.25, net. Lea Brothers & Co., Philadelphia and New York.

This book has stood the trial of a first edition, and has created a demand great enough to justify the author in revising and putting forth a second edition. The general arrangement of the first edition has been closely followed in the second, which was the result of a long experience in teaching.

The first edition established itself as a standard work, and now little is necessary to make the volume very popular wherever physiology is taught. The great number of details unnecessary to a clear understanding of the subject has been wisely omitted in the preparation of this book, and yet no important addition to our knowledge has been left out. The revision has covered all of the recent literature, and every advance that has been discovered of late years, bringing the work up to the present in a manner highly satisfactory to the profession. While much new matter has been added the number of pages of this edition is no greater than the first. The illustrations are the best, and as plentiful as in the other edition, and the price is notably lower. T. J. B.

